# Prevalence of Bactibilia in Patients Undergoing Cholecystectomy for Gallbladder Diseases: An Observational Study

**DOI:** 10.7759/cureus.97280

**Published:** 2025-11-19

**Authors:** Naveen Naik Mude, Shanmugam Dasarathan, Sudharsanan Sundaramurthi, Nagarajan Raj Kumar, Gopal Balasubramanian, Suresh Chilaka, Rakhi Biswas

**Affiliations:** 1 Surgery, Jawaharlal Institute of Postgraduate Medical Education and Research (JIPMER), Puducherry, IND; 2 Microbiology, Jawaharlal Institute of Postgraduate Medical Education and Research (JIPMER), Puducherry, IND

**Keywords:** antibiotic resistance, bactibilia, cholecystectomy, gallbladder disease, ssi

## Abstract

Introduction: Bactibilia, the presence of bacteria in bile, is a significant concern in patients undergoing cholecystectomy for gallbladder diseases. It may contribute to post-operative complications, influencing surgical outcomes. Despite its clinical relevance, data on the prevalence, microbial spectrum, and antibiotic resistance patterns of bactibilia in the Indian population remain limited. This study aimed to assess the prevalence of bactibilia, identify the predominant bacterial isolates, and evaluate their antimicrobial susceptibility patterns.

Methods: This cross-sectional study was conducted in a tertiary care center in South India. Bile samples were collected intraoperatively from 121 patients undergoing interval cholecystectomy after at least six weeks of conservative management of acute cholecystitis. Microbiological analysis included both aerobic and anaerobic cultures, as well as antibiotic susceptibility testing. Postoperative outcomes, including surgical site infections (SSIs), were documented over a 30-day follow-up period.

Results: Bactibilia was present in 57.9% of cases, with a higher prevalence in females (65.6%) and patients aged 51-60 years. The most common isolates were Escherichia coli (62.0%) and Acinetobacter baumannii (11.4%). Gram-negative bacteria exhibited high resistance to third-generation cephalosporins and fluoroquinolones but remained sensitive to amikacin and meropenem. Despite the presence of bactibilia, no postoperative SSI was observed, likely due to prophylactic antibiotic use.

Conclusion: A significant proportion of patients undergoing cholecystectomy had bactibilia, predominantly caused by E. coli and Acinetobacter. The study highlights emerging patterns of antimicrobial resistance, emphasizing the need for judicious antibiotic selection based on local antibiograms. Further research is warranted to refine antibiotic prophylaxis strategies in gallbladder surgery.

## Introduction

Bile is sterile under conditions of no external influx. The risk of bile infection increases in the presence of gallstones or biliary obstruction [1]. Bactibilia is a bacterial infection in the bile and is an important risk factor for post-operative complications. Incidence of complications due to bactibilia may increase the risk of adverse intra-operative and post-operative outcomes. The use of preoperative and postoperative antibiotics to mitigate infection is widely practiced despite a lack of sound bacteriologic and epidemiologic evidence [2]. The antibiogram, pathogens’ antibiotic resistance, and disease severity must be considered while deciding on antibiotic use [3]. Hence, this study was done to identify the spectrum of biliary infection in patients undergoing surgery for gall bladder diseases.

Assessing the bacteriological profile of bile in the gall bladder may provide greater insights into the role of infection in various gall bladder diseases in patients undergoing cholecystectomy. The bacteriological profile differs according to the patient’s demographic characteristics, and treating patients based on the results of studies from other populations may not be appropriate. Studies on bactibilia from the Indian population are sparse, and therefore, this study was done to help identify the bacterial profile associated with gall bladder disease in our population. The prevalence of common bacterial infections in gall bladder diseases can be studied, and thereby, prophylactic antibiotics can be tailored according to the subset of gall bladder diseases in the population.

On the other hand, surgical site infections (SSIs) are the third most common healthcare infection and are a serious clinical issue associated with morbidity and mortality, which pose severe economic demands on health resources [4]. SSIs are infections that occur up to 30 days after surgery or up to one year in patients with implants, affecting either the surgical incision or the deep tissues at the operation site. The incidence of SSIs following cholecystectomy is approximately 3 to 4% [5,6]. In the literature, few studies have reported the beneficial effects of antibiotic prophylaxis in cases of open cholecystectomy, but their impact in laparoscopic surgery is not clearly established. Therefore, the beneficial effects of the use of prophylactic antibiotics in cases of cholecystectomy, especially laparoscopic surgery, are yet to be established [7]. The objectives of the study were to determine the prevalence of bactibilia among patients undergoing interval cholecystectomy for acute cholecystitis, to identify the microbiological profile and antibiotic resistance patterns of bile isolates, and to evaluate the relationship between bactibilia and clinical or demographic factors such as age, gender, and comorbidities. The incidence of SSI following cholecystectomy was also estimated.

## Materials and methods

This was an observational cross-sectional study conducted in the Department of Surgery at a tertiary care institute in South India. The Institute’s Ethics Committee (IEC) approved the study (JIP/IEC/2019/280), and all the provisions of the Declaration of Helsinki were followed. After explaining the study's objectives and procedures, written informed consent for participation and publication was obtained from patients who agreed to participate in the study. The study included adult patients diagnosed with acute cholecystitis based on clinical, laboratory, and ultrasonographic criteria who underwent interval laparoscopic cholecystectomy after at least six weeks of conservative management during the study period from 2020 to 2022. Patients with chronic cholecystitis, biliary colic, prior biliary interventions such as endoscopic retrograde cholangiopancreatography (ERCP) or percutaneous drainage, recent antibiotic exposure within two weeks before surgery, or histopathological evidence of gallbladder malignancy were excluded from the study.

Study procedure

After recruitment, the baseline demographic characteristics and blood investigation reports of the patients were collected. An abdominal ultrasound was performed in all patients to assess the number of stones, gallbladder wall thickening, edema, and the presence of pericholecystic collection. Preoperative ultrasonography findings consistent with acute cholecystitis included gallbladder wall thickening (>3 mm), pericholecystic fluid collection, gallbladder distension, and a positive sonographic Murphy’s sign, as defined by the Tokyo Guidelines 2018. On the day of surgery, ceftriaxone was administered intravenously as antibiotic prophylaxis to all patients, and the intraoperative findings were recorded. All surgeries were performed by consultant surgeons or senior residents following standardized institutional protocols. Bile was aspirated intraoperatively from the gall bladder specimen after retrieval using a sterile syringe and needle for microbial culture. After specimen retrieval, the size of the gallbladder, the colour of the aspirated bile, and the number and type of stones were noted. Morphological features of the gallbladder and mucosal surface and irregularity were noted, and the gallbladder was sent for histopathological examination.

The samples were processed as per standard procedures, which include microscopy of the Gram-stained smear, aerobic and anaerobic cultures, and antibiotic susceptibility testing (AST). Susceptibility testing was performed using the Kirby-Bauer disk diffusion method. For anaerobic culture, the bile was inoculated into Robertson’s cooked meat (RCM) medium and transported to the microbiology laboratory within two hours at room temperature. The patients were examined for SSI at port sites on post-operative day three. The patients were followed up on postoperative days seven and 30, either in person or via telecommunication, to assess the presence of SSI. SSIs were defined according to the Centers for Disease Control and Prevention (CDC) criteria. Patients reporting local symptoms (pain, erythema, discharge, fever) were advised to attend for physical examination and wound evaluation.

Postoperative morbidities were noted according to the Clavien-Dindo classification [8]. The microbiological pattern of the organisms isolated from bile and the sensitivity pattern of the isolates were analyzed.

Statistical analysis

Categorical variables, such as gender and smoking history, were expressed as frequencies with proportions. Fisher’s exact test or Pearson’s chi-square test was used for statistical analysis. Continuous variables like age and BMI were represented as mean with standard deviation or median with interquartile range (IQR) as per the normality of the data. An unpaired Student's t-test or Mann-Whitney test was used to establish significant differences between the groups. The presence of bactibilia was reported as proportions. All statistical analyses were carried out at a 5% significance level, and a p-value of less than 0.05 was considered significant. Statistical tests were performed using SPSS Statistics version 19.0 (IBM Corp., Armonk, NY, USA).

## Results

A total of 121 consecutive patients aged >18 years were included in the study, of whom 61 were female (50.4%) and 60 were male (49.6%). The most common comorbidities were systemic arterial hypertension and diabetes mellitus. Patients with hypertension were 38 (31.4%), and those with diabetes mellitus were 32 (26.4%). There was no statistically significant difference in bacteria identification in bile samples from patients with and without comorbidities (hypertension, P=0.696; diabetes mellitus, P=0.535).

Among the participants, 64 (52.9%) had mild cholecystitis, and the remaining 57 (47.1%) had moderate cholecystitis as per the Tokyo guidelines. The incidences of bactibilia in mild and moderate cholecystitis were 60.1% and 54.4% respectively. The difference in the proportions between the two groups was not statistically significant (P=0.466). The prevalence of bactibilia was higher in females (40, 65.6%) than in males (30, 50%). The highest prevalence of bactibilia was observed in the age group of 51 to 60 years, i.e., 23 out of 30 (76.7%) in both females and males. The difference in the prevalence of bactibilia between males and females was not statistically significant (p=0.083).

Laparoscopic cholecystectomy was performed in 115 (95%) patients, of whom 64 (55.6%) had bile samples that showed bactibilia. Laparoscopic cholecystectomy was converted to open surgery in six (5%) patients due to technical difficulties. All bile samples from these patients showed bactibilia. The difference in proportions between the two groups was statistically significant (p=0.039).

In the study cohort, 114 (94.2%) stayed in the hospital for less than two days postoperatively, among whom 66 (57.9%) had bile samples with bacterial growth. Of the seven patients who stayed in the hospital for more than two days postoperatively, four (57.1%) patients had bactibilia. The difference in the proportions between the two groups was statistically insignificant (p=1.00) (Table 1). The culture status of the study population is shown in Table 2.

**Table 1:** Baseline Characteristics and Risk Factors for Bactibilia (N=121) *Fisher’s Exact Test,  †Pearson Chi-Square

Parameter	Negative culture (N,%)	Positive culture (N,%)	Total (N)	p value
Age group (years)				
<40	10 (35.7%)	18 (64.3%)	28	0.058*
41-50	17 (56.7%)	13 (43.3%)	30	
51-60	7 (23.3%)	23 (76.7%)	30	
61-70	15 (51.7%)	14 (48.3%)	29	
>70	2 (50%)	2 (50%)	4	
Gender				
Male	30 (50%)	30 (50%)	60	0.083 ^†^
Female	21 (34.4%)	40 (65.6%)	61	
Grade of cholecystitis				
Mild	25 (39.1%)	39 (60.9%)	64	0.466 ^†^
Moderate	26 (45.6%)	31 (54.4%)	57	
Comorbidities				
Diabetes Present	12 (37.5%)	20 (62.5%)	32	0.535 ^†^
Diabetes Absent	39 (43.8%)	50 (56.2%)	89	
Hypertension Present	17 (44.7%)	21 (55.3%)	38	0.696^ †^
Hypertension Absent	34 (41.0%)	49 (59.0%)	83	
Approach of surgery				
Laparoscopic	51 (44.3%)	64 (55.7%)	115	0.039*
Lap converted to open	0 (0%)	6 (100%)	6	
Post-operative length of stay in hospital				
Less than 2days	48 (42.1%)	66 (57.9%)	114	1.0*
More than 2days	3 (42.9%)	4 (57.1%)	7	

**Table 2:** Microbial Isolates from Aerobic and Anaerobic Cultures (N=121)

Parameter	Total isolates (N)
Aerobic culture (Yield: 79 organisms from 70 positive cultures)	
Facultative aerobes/anaerobes	77
Obligate aerobes	2
Anaerobic culture (Yield: 4 organisms from 4 positive cultures)	
Obligate anaerobes (Bacteroides fragilis)	4
Gram Stain Pattern (N=79)	
Gram-negative bacteria	72
Gram-positive bacteria	7

Out of 121 samples, in 51 (42.1%) samples there was no bacterial growth (Sterile), and 70 (57.9%) samples were bile culture positive. Out of 70 aerobic culture-positive samples, a single bacterial species (monomicrobial growth) was identified in 61 cases (87.1%), and nine cases (12.9%) showed simultaneous growth of two different bacteria (polymicrobial growth).

The separately sent anaerobic culture of the samples grew organisms in four cases. Therefore, a total of 83 bacterial isolates were identified in the positive cultures: 79 from aerobic cultures and four from anaerobic cultures. Out of 79 aerobic microbes, 72 (91.1%) were Gram-negative strains, and seven (8.9%) were Gram-positive strains. The isolates from aerobic culture in the study population are depicted in Table 3.

**Table 3:** Microbial isolates from aerobic culture in the study population (N=79)

Isolate	Frequency (N)	Percentage (%)
Gram-negative isolates		
Escherichia coli	49	62.0%
Acinetobacter baumannii	9	11.4%
Enterobacter cloacae	6	7.6%
Klebsiella pneumoniae	5	6.3%
Pseudomonas aeruginosa	2	2.5%
Enterobacter asburiae	1	1.3%
Gram-positive isolates		
Enterococcus faecium	5	6.3%
Enterococcus faecalis	2	2.5%

Out of 79 microorganisms from the aerobic cultures, Escherichia coli (62%) and Acinetobacter baumannii (11.4%) were the most prevalent Gram-negative isolates. Enterococcus faecium was the most common Gram-positive microbe isolated, followed by Enterococcus faecalis.

Of the 121 samples processed for anaerobic culture, only four (3.3%) grew Bacteroides fragilis, a Gram-negative obligate anaerobe. A total of 43 (35.5%) gallbladder samples had pigment/ mixed gallstones, and 14 (11.6%) gallbladder samples had cholesterol stones. Twenty-one (48.8%) out of 43 patients with pigment/ mixed type gallstones and five (35.7%) out of 14 patients with cholesterol gallstones had negative/sterile cultures.

Antibiotic sensitivity pattern of aerobic isolates in the study population is summarized in Table 4.

**Table 4:** Antibiotic sensitivity pattern in study population (N=121; 79 isolates of aerobes and facultative anaerobes)

Antibiotic sensitivity pattern	Sensitive (N,%)	Resistant (N,%)
Gram-negative isolates (72)		
Amikacin	52 (72.2%)	20 (27.8%)
Cefperazone sulbactum	41 (56.9)	31 (43.1%)
Ceftazidime	16 (22.2%)	56 (77.8%)
Ceftriaxone	14 (19.4%)	58 (80.6%)
Ciprofloxacin	16 (22.2%)	56 (77.8%)
Colistin	38 (49.4%)	39 (50.6%)
Meropenam	46 (63.9)	26 (36.1%)
Tazobactum piperacillin	42 (58.3%)	30(41.7%)
Gram-positive isolates (7)		
Ampicillin	5 (71.4%)	2 (28.6%)
Vancomycin	7 (100%)	0 (0%)
Linezolid	7 (100%)	0 (0%)
Teicoplanin	7 (100%)	0 (0%)
Tetracycline	7 (100%)	0 (0%)

**Figure 1:**
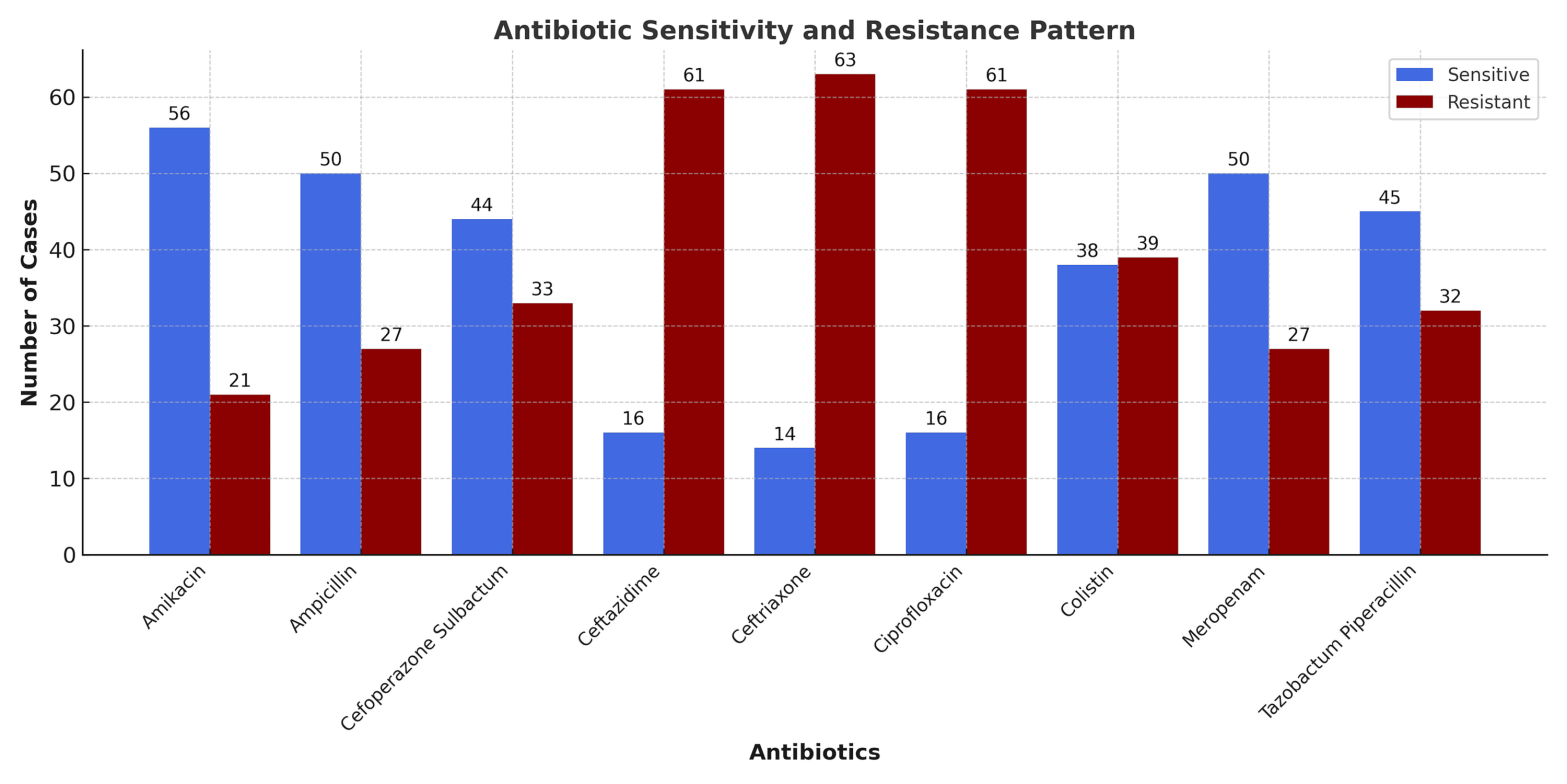
The antibiotic sensitivity and resistance pattern of cultured micro-organisms.

Out of the Gram-negative aerobic isolates, highest sensitivity was observed for amikacin 56 (72.7%) followed by meropenam 50 (63.9%), piperacillin-tazobactum 45 (58.4%) and those microbes showed highest resistance to third-generation cephalosporins [ceftriaxone 63 (81.8%), ceftazidime 61 (79.2%)] followed by fluoroquinolones 61 (79.2%) (Figure 1).

E. coli, the commonest organism isolated, was most sensitive to amikacin, followed by meropenem and piperacillin-tazobactam. Acinetobacter pathogens showed high resistance to all the antibiotics we used in our study. Klebsiella showed the highest sensitivity to piperacillin-tazobactam and highest resistance to amikacin, followed by β-lactams and third-generation cephalosporins.

All collected samples were processed for anaerobic culture, of which four samples grew anaerobic microbes; all four cultures reported Bacteroides fragilis. Out of these four isolates, three were sensitive to metronidazole. On follow-up, none of the patients in our study had an SSI at 30 days.

## Discussion

Gallbladder-related diseases are more common in Western populations. In India, a high incidence of gallbladder diseases, especially acute cholecystitis and cholelithiasis, is observed in the Northern states [9]. The present study aimed to determine the prevalence of bacterial infection in bile, antibiotic sensitivity patterns, and postoperative complications in the South Indian population. In this study, demographic factors such as age, gender, BMI, ASA class, comorbidities, significant personal history, diagnosis, severity grade, type of gallstones, duration of procedure, intraoperative bile spillage, length of hospital stay, and postoperative complications, including SSIs, were not statistically significant with the presence of bactibilia. The Tokyo Guidelines 2018 recommend obtaining bile cultures, particularly in moderate to severe (Grade II and III) cases of acute cholecystitis; however, real-world adherence to this recommendation has been reported to be suboptimal [10]. Bactibilia is common in high-risk patients, including those of advanced age or with acute cholecystitis, biliary obstruction, choledocholithiasis, and cholangitis [11]. In elective cholecystectomy cases, the incidence of bactibilia ranges from 9% to 42%, while in acute cholecystitis, it ranges from 35% to 65% [12,13]. In our study, bactibilia was observed in 57.85% of cases, comparable to previous studies.

Everhart et al. reported that North American Indians have the highest prevalence of cholelithiasis, affecting 64.1% of women and 29.5% of men. Cholesterol gallstones are predominant in developed Western countries, whereas brown pigment stones are more common in Asia. Very few reports exist on the prevalence of biliary infection in South Indian patients with gallstone disease, where mixed and pigment gallstones predominate, consistent with our findings [3,14]. In our study, a high prevalence of bacterial infection in gallstone disease patients (70 out of 121 cases, 57.9%) was also observed in other studies.

Gallbladder diseases in Indians are known to occur earlier, particularly in middle-aged, obese, multiparous women. The incidence of gallstones increases with age, particularly after the age of 40 [15]. In our study, culture-positive cases were most common in the 51-60-year age group, with a mean age of 51.50 ± 13.32 years. Though not statistically significant, female gender was the most common risk factor for bactibilia, consistent with previous studies, with females accounting for 57.14% of cases.

Mahafzah AM et al. concluded that bactibilia is influenced by age, gender, preoperative biliary tract interventions, associated morbidities, and complicated gallbladder disease but does not impact postoperative septic complications. They reported that age and preoperative biliary tract intervention were the only significant predictors of bactibilia. In high-risk patients with obstructive biliopathy, biliary tract surgery, advanced age, and cholangitis, bactibilia is a common finding [16]. Bang CS et al. reported that diabetic patients are more likely to develop gallbladder diseases, particularly cholecystitis [17]. In our study, 28.6% of diabetic patients had bactibilia. However, there was no statistically significant difference in bactibilia between patients with and without comorbidities. The absence of a statistically significant association between comorbidities and bactibilia may be related to the limited sample size and smaller proportion of patients with diabetes or hypertension, reducing the statistical power to demonstrate a difference.

Den Hoed PT et al. reported more postoperative complications in gallstone diseases, with approximately 45% of polymicrobial isolates associated with cholelithiasis. It is crucial to remove stones before starting antibiotic therapy because they act as a nidus for infection. Moreover, calculi promote chronic infection due to stasis, leading to increased secondary bile acid formation, which causes constant irritation and predisposes to biliary infection [18]. Our study showed a predominance of gram-negative infections in the biliary tract. The most commonly isolated microorganisms were intestinal flora, comparable with previous reports. Escherichia coli (62.0%) and Acinetobacter (11.4%) were the predominant Gram-negative pathogens causing bactibilia in our study.

Antibiotic sensitivity patterns have great clinical and epidemiological significance. Understanding bacterial types, resistance profiles, and polymicrobial cultures helps in selecting the most appropriate antimicrobial therapy. In many randomized studies, there was no significant difference in postoperative complications between patients who received prophylactic antibiotics and those who did not [19]. Our study found increased resistance rates for ceftriaxone (80.6%), ciprofloxacin (77.8%), and ceftazidime (77.8%). Penicillin-resistant organisms (ampicillin) accounted for 28.6%, while resistance to amikacin (27.8%) and meropenem (36.1%) was comparatively lower. Increased susceptibility of gram-negative isolates to aminoglycosides (amikacin) and increased resistance to third-generation cephalosporins and fluoroquinolones were observed, highlighting a shift in antibiotic susceptibility patterns. Although a direct comparison with the institutional antibiogram was not performed, our findings are consistent with local microbiology reports indicating increasing resistance to third-generation cephalosporins and fluoroquinolones, suggesting that the observed pattern reflects broader regional antimicrobial trends. The antibiotic susceptibility patterns observed reflect the local microbial profile in our study population, with notable resistance to commonly used cephalosporins. However, without specific MDR or ESBL classification in our data, broader comparisons with other studies were avoided. 

Based on bile culture results, carbapenems (meropenem), beta-lactam antibiotics (sulbactam), glycopeptides (vancomycin), penicillins (ampicillin), and aminoglycosides (amikacin) were the most preferable antibiotics. However, their clinical implications remain uncertain. Notably, approximately 60% of strains were resistant to commonly used prophylactic antibiotics, including ceftriaxone, ciprofloxacin, and ceftazidime. The high resistance rate (>80%) to ceftriaxone observed in our study highlights the need for periodic review and revision of institutional antibiotic prophylaxis protocols based on updated local antibiogram data.

A prospective cohort study of acute cholecystitis patients reported that 16.7% of E. coli isolates produced extended-spectrum beta-lactamases (ESBL) [20]. However, ESBL prevalence varies widely by region: 31.2% in Germany, 70.0% in Korea, and 66% in India [21-23]. Notably, several studies have shown a significantly higher risk of postoperative sepsis in patients with ESBL-producing bactibilia [24]. In our study, none of the patients developed postoperative complications. Several randomized studies in the literature have reported no significant difference in postoperative complications between patients who received prophylactic antibiotics and those who did not. Choudhary A et al. reported that antibiotic prophylaxis, irrespective of bile culture results, did not affect complication rates [25]. Similarly, Gurusamy KS et al. concluded that prophylactic antibiotics did not reduce the incidence of SSIs, regardless of the surgical procedure [26]. In laparoscopic cholecystectomy, the role of prophylactic antibiotics in low-risk patients is controversial, though some studies recommend them for high-risk patients [11]. Chang WT et al. reported no significant benefit of prophylactic antibiotics in preventing SSIs and postoperative complications in elective laparoscopic cholecystectomy [27]. Studies by Rodriguez et al. and Malik SA et al. found no role for antibiotic prophylaxis in preventing SSIs [28,29]. Yan RC et al. claimed that the pathogenic strains responsible for infection vary among patients, regardless of prophylaxis [30].

Bogdanic B et al. reported SSI rates of 6.06% in open cholecystectomy, 0.60% in laparoscopic cases, and 17.9% in converted cases, with an overall incidence of 1.44% [4]. In our study, all patients received preoperative prophylactic antibiotics (ceftriaxone 1 g) as a standard protocol, and none developed postoperative SSI. Darkahi et al. demonstrated that bactibilia was the only significant factor associated with postoperative complications, while Mahafzah et al. reported that bactibilia did not affect postoperative complications [11,16]. In this study, no patient developed postoperative complications, which contradicts previous studies. Asai K et al. concluded that broad-spectrum antimicrobials should be administered perioperatively, even for mild cholecystitis cases [10]. Taken together, routine antibiotic prophylaxis is not required for all gallbladder disease patients, but broad-spectrum antibiotic prophylaxis is recommended for those with preoperative or intraoperative risk factors. Although no surgical site infections were observed in our study cohort, this finding cannot be directly attributed to the use of prophylactic antibiotics, given the observational nature of the study. Other contributing factors, such as low-risk patients, standardized aseptic technique, short operative duration, and postoperative wound care practices, may also have influenced the outcome. 

One of the major limitations of our study is its single-centre design, which may limit the generalizability of the findings to a broader population. These findings primarily apply to patients undergoing interval laparoscopic cholecystectomy for acute cholecystitis in similar tertiary care settings and may not be generalizable to emergency or open cholecystectomy cases. The sample size, while sufficient for analysis, may not capture the full spectrum of variability in bacteriological profiles and antibiotic resistance patterns across different regions. Although the study provides valuable insights into the microbiological profile of bile in patients undergoing interval cholecystectomy, the absence of statistically significant associations for certain clinical variables may be attributed to the limited sample size and small subgroup numbers rather than a true lack of relationship. Larger, adequately powered studies are needed to further explore these potential associations. Additionally, the lack of long-term follow-up beyond 30 days precludes the assessment of late postoperative complications or recurrence of infections. Further, while antibiotic susceptibility testing was conducted, molecular-level resistance mechanisms were not explored, thereby limiting a deeper understanding of the emerging antimicrobial resistance patterns. As all patients received preoperative prophylactic antibiotics as per institutional protocol, it was not possible to evaluate the actual impact of prophylactic antibiotics on post-operative infection rates. Our study included mostly laparoscopic cases, with only a few conversions to open cholecystectomy, limiting the ability to assess outcomes in open surgery cases. However, it is well established that the SSI rate in laparoscopic surgery is relatively lower than that of open cholecystectomy.

## Conclusions

Our study provides valuable insights into the prevalence of bacterial infection in bile and the antibiotic susceptibility patterns of common pathogens causing bactibilia. The findings confirm that bactibilia is frequently associated with gallbladder disease, particularly in high-risk groups such as older individuals and female patients. Escherichia coli and Acinetobacter baumannii were the predominant pathogens, with significant resistance to third-generation cephalosporins and fluoroquinolones, highlighting the growing concern of antimicrobial resistance. Although prophylactic antibiotics are widely used, their role in preventing SSIs remains debatable, particularly in laparoscopic procedures where the incidence of SSIs is low. This study underscores the need for judicious antibiotic selection based on local antibiograms to combat emerging resistance patterns effectively. Further research is warranted on the role of bile cultures and targeted antibiotic use in different surgical settings, such as elective versus emergency cholecystectomy, laparoscopic versus open procedures, and in patients with acute cholecystitis, choledocholithiasis, or obstructive jaundice. Future multicenter studies focusing on benign gallbladder diseases such as gallstone disease and acute or chronic cholecystitis, with standardized data collection and stratification according to local antibiotic policies, along with long-term follow-up and molecular-level resistance profiling, would provide a more comprehensive understanding of the evolving bacterial landscape.

## References

[1] Yun SP, Seo HI (2018). Clinical aspects of bile culture in patients undergoing laparoscopic cholecystectomy. Medicine (Baltimore).

[2] Cueto-Ramos R, Hernández-Guedea M, Pérez-Rodríguez E, Reyna-Sepúlveda F, Muñoz-Maldonado G (2017). Incidence of bacteria from cultures of bile and gallbladder wall of laparoscopic cholecystectomy patients in the University Hospital “Dr. José Eleuterio González”. Cir Cir Engl Ed.

[3] Pavithra S, Rao U, Mohan P, Venkataraman J (2010). Bactibilia in pigment gallstone disease: a report from the Indian subcontinent. Dig Liver Dis.

[4] Bogdanic B, Bosnjak Z, Budimir A (2013). Surveillance of surgical site infection after cholecystectomy using the hospital in Europe link for infection control through surveillance protocol. Surg Infect (Larchmt).

[5] Biscione FM, Couto RC, Pedrosa TM, Neto MC (2007). Comparison of the risk of surgical site infection after laparoscopic cholecystectomy and open cholecystectomy. Infect Control Hosp Epidemiol.

[6] (2004). National Nosocomial Infections Surveillance (NNIS) System Report, data summary from January 1992 through June 2004, issued October 2004. Am J Infect Control.

[7] Zhou H, Zhang J, Wang Q, Hu Z (2009). Meta-analysis: antibiotic prophylaxis in elective laparoscopic cholecystectomy. Aliment Pharmacol Ther.

[8] Dindo D, Demartines N, Clavien PA (2004). Classification of surgical complications: a new proposal with evaluation in a cohort of 6336 patients and results of a survey. Ann Surg.

[9] Ebert EC, Nagar M, Hagspiel KD (2010). Gastrointestinal and hepatic complications of sickle cell disease. Clin Gastroenterol Hepatol.

[10] Asai K, Watanabe M, Kusachi S (2012). Bacteriological analysis of bile in acute cholecystitis according to the Tokyo guidelines. J Hepatobiliary Pancreat Sci.

[11] Darkahi B, Sandblom G, Liljeholm H, Videhult P, Melhus Å, Rasmussen IC (2014). Biliary microflora in patients undergoing cholecystectomy. Surg Infect (Larchmt).

[12] Galili O, Eldar S Jr, Matter I, Madi H, Brodsky A, Galis I, Eldar S Sr (2008). The effect of bactibilia on the course and outcome of laparoscopic cholecystectomy. Eur J Clin Microbiol Infect Dis.

[13] Lee SW, Yang SS, Chang CS, Yeh HJ (2009). Impact of the Tokyo guidelines on the management of patients with acute calculous cholecystitis. J Gastroenterol Hepatol.

[14] Chuang SC, Lee KT, Chang WT, Wang SN, Kuo KK, Chen JS, Sheen PC (2004). Risk factors for wound infection after cholecystectomy. J Formos Med Assoc.

[15] Stinton LM, Shaffer EA (2012). Epidemiology of gallbladder disease: cholelithiasis and cancer. Gut Liver.

[16] Mahafzah AM, Daradkeh SS (2009). Profile and predictors of bile infection in patients undergoing laparoscopic cholecystectomy. Saudi Med J.

[17] Bang CS, Yoon JH, Kim YJ (2014). Clinical impact of body mass index on bactibilia and bacteremia. BMC Gastroenterol.

[18] Den Hoed PT, Boelhouwer RU, Veen HF, Hop WC, Bruining HA (1998). Infections and bacteriological data after laparoscopic and open gallbladder surgery. J Hosp Infect.

[19] Paterson DL, Ko WC, Von Gottberg A (2004). International prospective study of Klebsiella pneumoniae bacteremia: implications of extended-spectrum beta-lactamase production in nosocomial Infections. Ann Intern Med.

[20] Coccolini F, Sartelli M, Catena F (2015). Antibiotic resistance pattern and clinical outcomes in acute cholecystitis: 567 consecutive worldwide patients in a prospective cohort study. Int J Surg.

[21] Reuken PA, Torres D, Baier M (2017). Risk factors for multi-drug resistant pathogens and failure of empiric first-line therapy in acute cholangitis. PLoS One.

[22] Kwon JS, Han J, Kim TW (2014). Changes in causative pathogens of acute cholangitis and their antimicrobial susceptibility over a period of 6 years. Korean J Gastroenterol.

[23] Shenoy SM, Shenoy S, Gopal S, Tantry BV, Baliga S, Jain A (2014). Clinicomicrobiological analysis of patients with cholangitis. Indian J Med Microbiol.

[24] Pagani MA Jr, Dolfini PM, Trazzi BF (2023). Incidence of bacteriobilia and the correlation with antibioticoprophylaxis in low-risk patients submitted to elective videolaparoscopic cholecystectomy: a randomized clinical trial. Antibiotics (Basel).

[25] Choudhary A, Bechtold ML, Puli SR, Othman MO, Roy PK (2008). Role of prophylactic antibiotics in laparoscopic cholecystectomy: a meta-analysis. J Gastrointest Surg.

[26] Gurusamy KS, Koti R, Wilson P, Davidson BR (2013). Antibiotic prophylaxis for the prevention of methicillin-resistant Staphylococcus aureus (MRSA) related complications in surgical patients. Cochrane Database Syst Rev.

[27] Chang WT, Lee KT, Chuang SC, Wang SN, Kuo KK, Chen JS, Sheen PC (2006). The impact of prophylactic antibiotics on postoperative infection complication in elective laparoscopic cholecystectomy: a prospective randomized study. Am J Surg.

[28] Rodríguez-Sanjuán JC, Casella G, Antolín F (2013). How long is antibiotic therapy necessary after urgent cholecystectomy for acute cholecystitis?. J Gastrointest Surg.

[29] Malik SA, Yasin MA, Nasreen G (2009). Single and simple antibiotic prophylaxis for elective cholecystectomy. J Coll Physicians Surg Pak.

[30] Yan RC, Shen SQ, Chen ZB, Lin FS, Riley J (2011). The role of prophylactic antibiotics in laparoscopic cholecystectomy in preventing postoperative infection: a meta-analysis. J Laparoendosc Adv Surg Tech A.

